# Painful penile mass as an initial manifestation of systemic diffuse large B-cell lymphoma: a case report

**DOI:** 10.3389/fmed.2026.1882276

**Published:** 2026-08-17

**Authors:** Min-Jung Jan, Chia Hao Liang, Ming-Che Ou, Tang-Yi Tsao, Yi-Sheng Lin, Yen-Chuan Ou, Min-Che Tung, Chao-Yu Hsu

**Affiliations:** 1 Department of Medical Education, Tungs’ Taichung MetroHarbor Hospital, Taichung, Taiwan; 2 Department of Medical Education, Dalin Tzu Chi Hospital, Dalin, Taiwan; 3 Department of Hematology and Oncology, Tungs’ Taichung MetroHarbor Hospital, Taichung, Taiwan; 4 Department of Pathology, Tungs’ Taichung MetroHarbor Hospital, Taichung, Taiwan; 5 Division of Urology, Department of Surgery, Tungs’ Taichung MetroHarbor Hospital, Taichung, Taiwan

**Keywords:** diffuse large B-cell lymphoma, extranodal lymphoma, FDG PET/CT, penile lymphoma, penile mass, R-CHOP

## Abstract

**Background:**

Diffuse large B-cell lymphoma (DLBCL) involving the penis is an exceptionally rare extranodal malignancy and is frequently misdiagnosed as penile carcinoma or infectious conditions because of its nonspecific clinical presentation.

**Case presentation:**

A 58-year-old man presented with a progressively enlarging painful penile mass for 3 months that was refractory to empirical antibiotic therapy. Ultrasound-guided core biopsy of the penile shaft mass revealed diffuse large B-cell lymphoma, centroblastic type, non–germinal center B-cell subtype with MYC and BCL-2 double expression. Fluorodeoxyglucose positron emission tomography/computed tomography (FDG PET/CT) demonstrated intense uptake in the penis with additional gastric involvement confirmed by biopsy, consistent with Lugano stage IV disease. The patient received curative-intent systemic chemo-immunotherapy with Rituximab plus Cyclophosphamide, Doxorubicin, Vincristine, and Prednisone (R-CHOP) and subsequently achieved complete metabolic remission on follow-up FDG PET/CT.

**Conclusion:**

This case highlights an unusual presentation of systemic DLBCL as a painful penile mass and underscores the pivotal role of FDG PET/CT in detecting occult extranodal involvement, accurately staging disease, and guiding appropriate management.

## Introduction

Diffuse large B-cell lymphoma (DLBCL) is the most common subtype of non-Hodgkin lymphoma and frequently involves extranodal sites (1). Involvement of the penis is rare, with only a few dozen cases reported worldwide (1). Reported clinical presentations include painless penile masses, swelling, ulceration, or urinary symptoms. Notably, a recent literature review found that approximately one-third of reported cases initially presented as painless penile lesions, whereas clinically significant pain is relatively uncommon (1). Because penile masses are far more commonly attributed to epithelial malignancies or infectious etiologies, lymphoma involving the penis is seldom considered during initial evaluation, often leading to delayed diagnosis and inappropriate management (2, 3).

In addition to histopathological confirmation, FDG PET/CT plays a pivotal role in the initial evaluation of DLBCL by accurately staging disease, detecting occult extranodal involvement, and guiding treatment planning (4–6). The clinical significance of recognizing this entity lies in its fundamentally different treatment strategy. Unlike penile carcinoma, which is often managed surgically, penile diffuse large B-cell lymphoma is primarily treated with systemic chemotherapy with organ-preserving intent (1, 2). We present a rare case of systemic DLBCL initially presenting as a painful penile mass with advanced-stage disease, highlighting important diagnostic pitfalls and clinical implications.

## Case presentation

A 58-year-old man with a medical history of chronic obstructive pulmonary disease, type 2 diabetes mellitus, and hypertension presented to the outpatient clinic with penile pain and a palpable mass that had worsened over 1 week. He reported first noticing a small lump at the base of the penis approximately 3 months prior, which gradually enlarged and became increasingly painful. At the initial outpatient visit, empirical antibiotic therapy was prescribed. At follow-up 1 week later, the patient reported no clinical improvement, with persistent pain and progressive enlargement of the mass. He denied dysuria, hematuria, fever, weight loss, or night sweats.

**Figure 1:**
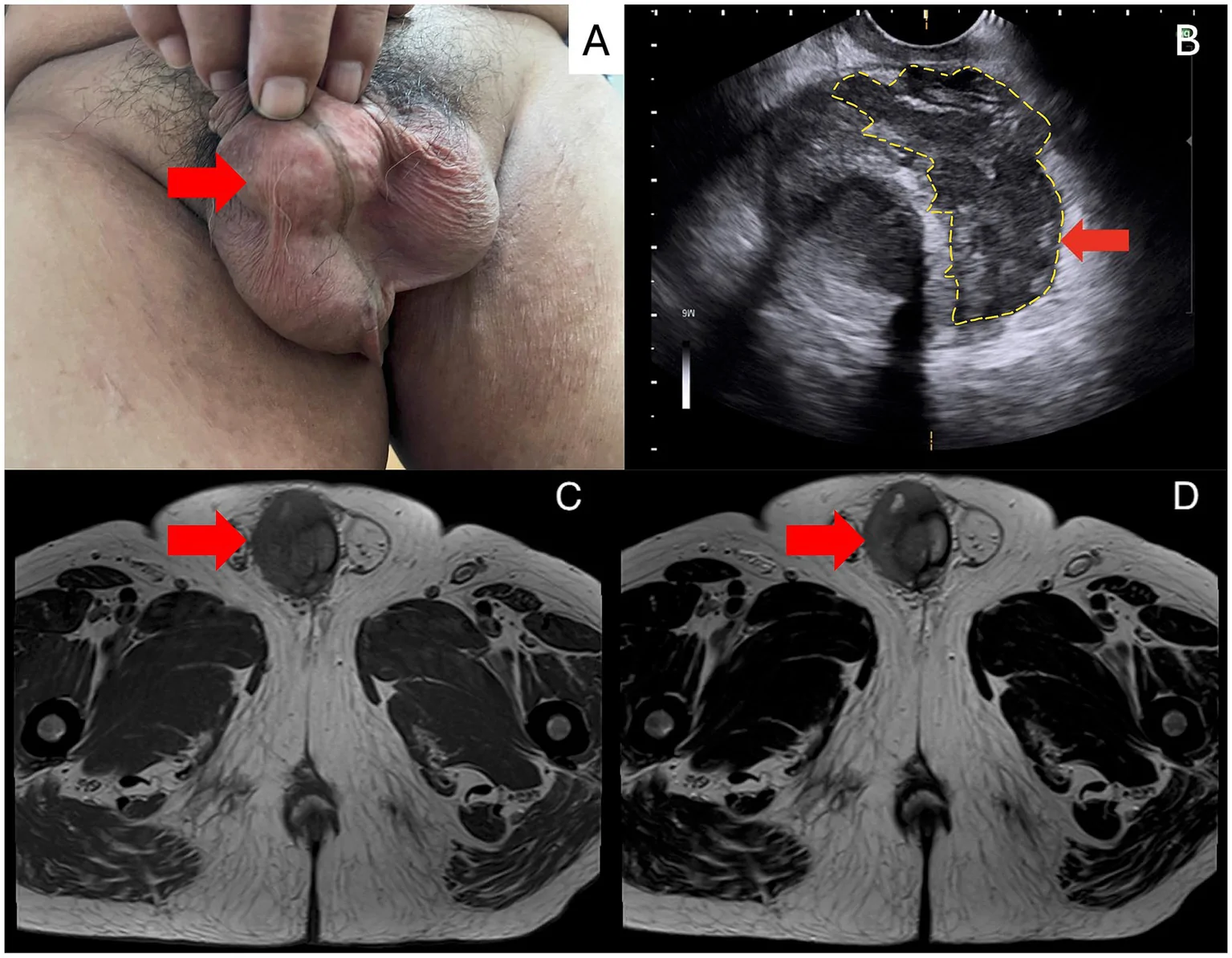
**(A)** Clinical photograph showing diffuse penile enlargement with a prominent mass predominantly involving the right side of the penile shaft (arrow), corresponding to the ultrasound-guided biopsy site. **(B)** Penile ultrasonography demonstrates a heterogeneous mass involving the corpora cavernosa and confined within the tunica albuginea, without definite invasion of the urethra. **(C,D)** Pelvic MRI shows an 8 × 4.6 cm mass involving the right corpus cavernosum and tunica albuginea, hypointense on T1- and T2-weighted images with restricted diffusion, suspicious for malignancy. No enlarged pelvic lymph nodes are seen.

Physical examination revealed a firm, tender mass involving the entire penile shaft (Figure 1A), without overlying skin ulceration or signs of acute infection. No palpable inguinal lymphadenopathy was identified. Laboratory investigations, including complete blood count and renal and hepatic function tests, were within normal limits. Based on the patient’s clinical presentation, the differential diagnoses included penile inflammation, penile squamous cell carcinoma, Peyronie’s disease, and benign or malignant soft tissue neoplasm.

Penile ultrasonography demonstrated a heterogeneous lesion confined within the tunica albuginea, without definite invasion of the urethra (Figure 1B). For further local staging, pelvic magnetic resonance imaging was subsequently performed and revealed an approximately 8 × 4.6 cm soft tissue mass involving the right corpus cavernosum and tunica albuginea, showing hypointense signals on T1- and T2-weighted images and restricted diffusion, suggestive of a malignant process. (Figures 1C,D). Given the atypical presentation and poor response to antibiotics, an ultrasound-guided core biopsy was performed through the irregular right lateral aspect of the penile shaft (Figure 1B).

**Figure 2:**
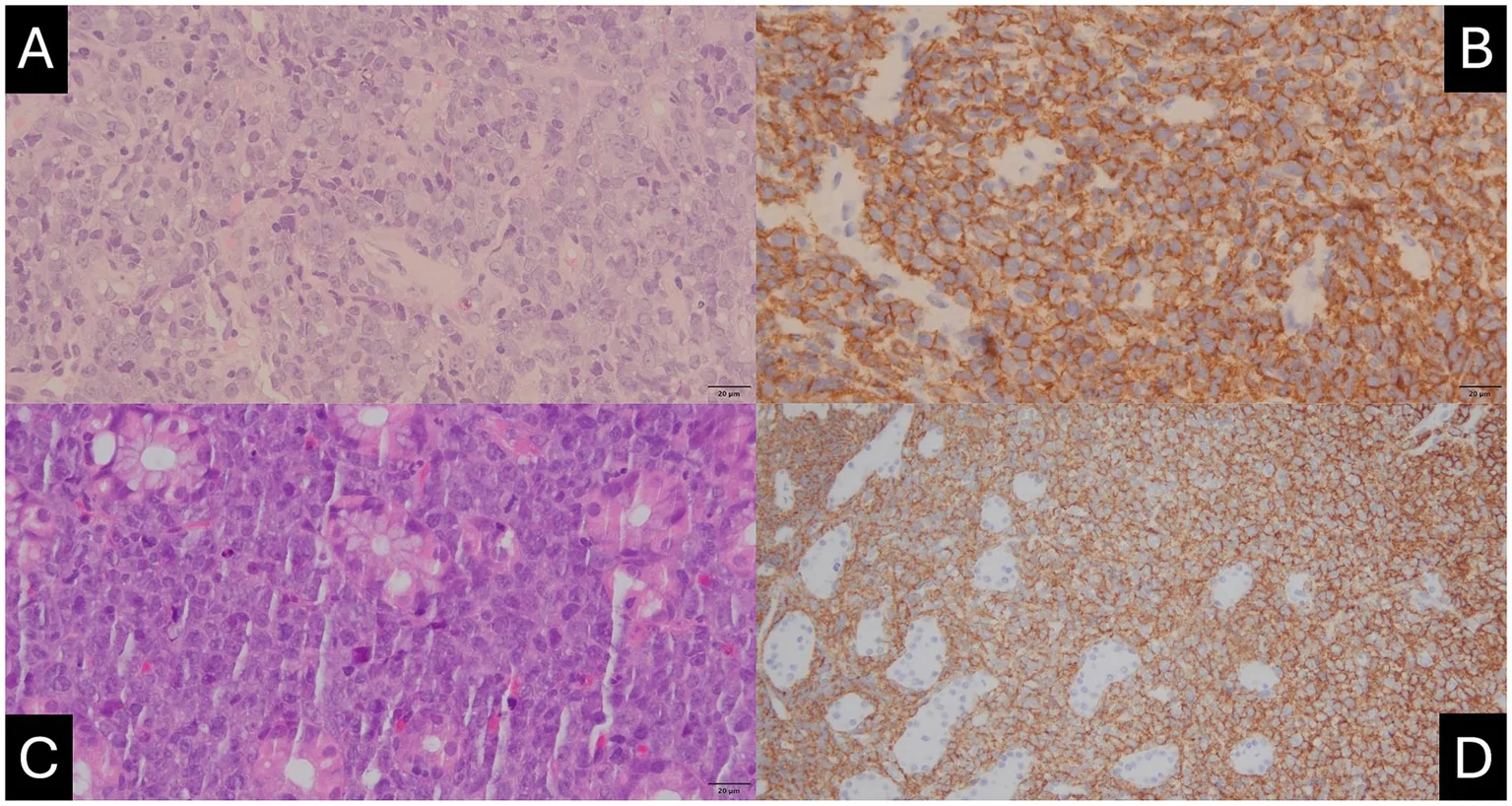
Histopathological findings of the penile lesion. **(A)** In penis mass biopsy specimen, the lymphoma cells with large round, oval, or irregular nuclei, multiple punctate and occasional large central nucleoli (hematoxylin–eosin stain x 400) **(B)** In penis mass biopsy specimen, the lymphoma cells show diffusely positive for CD20 with membranous pattern (Immunohistochemical stain x 200). **(C)** In gastric biopsy specimen, the lymphoma cells with large round, oval, or irregular nuclei, multiple punctate nucleoli diffusely infiltrate in between the gastric glands causing wide separation and disrupting the native architecture (hematoxylin–eosin stain x 400) **(D)** In gastric biopsy specimen, the lymphoma cells show diffusely positive for CD20 with membranous pattern (Immunohistochemical stain x 200).

Histopathological examination of the penile core biopsy revealed diffuse infiltration of large atypical lymphoid cells with abundant cytoplasm, round to oval nuclei, prominent nucleoli, and frequent mitotic figures (Figure 2). Immunohistochemical staining showed that the tumor cells were positive for CD20, BCL-2 (>95%), BCL-6 (>90%), MUM1 (>90%), and MYC (~60%), and negative for CD10, CD5, CD23, cyclin D1, and CD30. CD3 highlighted background reactive T lymphocytes. Based on the immunophenotypic profile (CD10−, BCL6+, MUM1+), the lymphoma was classified as a non–germinal center B-cell (non-GCB) subtype according to the Hans algorithm. Co-expression of MYC (~60%) and BCL-2 (>95%) was consistent with double expression lymphoma (DEL). Ki-67 proliferation index was 90%. *In situ* hybridization for Epstein–Barr virus-encoded RNA (EBER) was negative. These findings supported the diagnosis of diffuse large B-cell lymphoma (DLBCL), centroblastic type, non-GCB subtype.

**Figure 3:**
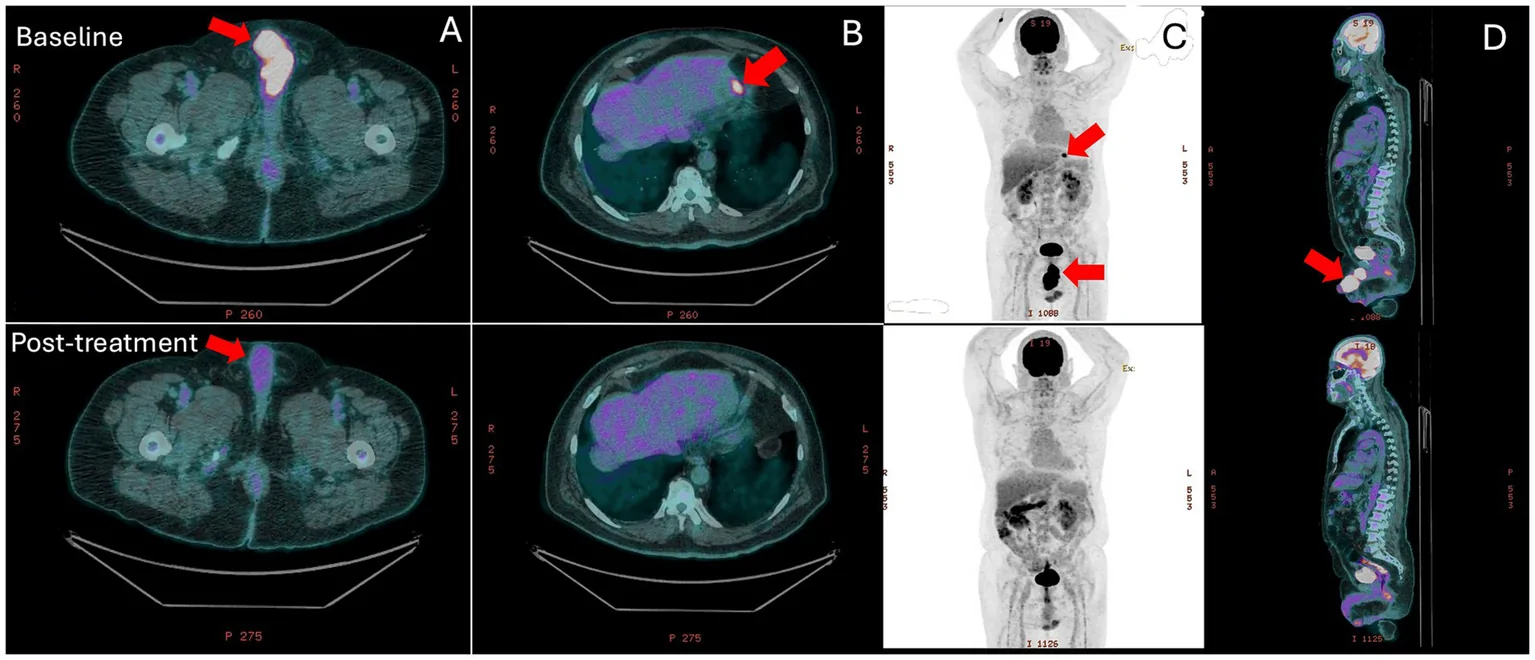
Comparison of baseline and post-treatment FDG PET/CT findings. Baseline images (upper row) and follow-up images after the fourth cycle of R-CHOP therapy (lower row) are shown: **(A)** Axial fused PET/CT images demonstrate intense FDG uptake in the penile lesion at baseline (SUVmax 34.28 early and 36.76 delayed), which markedly decreased on follow-up (SUVmax 2.95), consistent with significant metabolic response (Deauville score 5 → 3). **(B)** Axial fused PET/CT images of the stomach show an additional FDG-avid lesion at baseline (SUVmax 6.82 early and 7.96 delayed), with markedly reduced uptake after treatment (SUVmax 2.69–3.23), indicating interval metabolic regression (Deauville score 3 → 2). **(C)** Whole-body maximum intensity projection images demonstrate focal FDG uptake in the penis and stomach at baseline, with marked reduction of previously noted hypermetabolic lesions and no newly identified FDG-avid lesions on follow-up. **(D)** Sagittal fused PET/CT images show intense FDG uptake in the penile mass at baseline, with markedly decreased metabolic activity after treatment, consistent with treatment response.

Subsequent fluorodeoxyglucose positron emission tomography/computed tomography (FDG PET/CT) demonstrated intense metabolic activity in the penile lesion (SUVmax 36.76, Deauville score 5) (Figures 3A,D), along with additional FDG-avid lesions in the stomach (Figure 3B). Upper gastrointestinal endoscopy with biopsy of the lower body of the stomach subsequently confirmed gastric involvement by diffuse large B-cell lymphoma, characterized by sheets of large atypical lymphoid cells expressing CD20 and BCL-2, and lacking CD3 and cyclin D1 expression.

Based on these findings, the disease was classified as malignant lymphoma, diffuse large B-cell, centroblastic type, involving the penis and stomach, Lugano classification stage IV, with an ECOG performance status of 1 and an International Prognostic Index score of 2. Curative-intent systemic chemo-immunotherapy with R-CHOP was initiated.

Following initiation of systemic chemo-immunotherapy, the patient received 6 cycles of Rituximab plus Cyclophosphamide, Doxorubicin, Vincristine, and Prednisone (R-CHOP). The treatment was generally well tolerated, with no significant adverse effects reported.

FDG PET/CT performed after the fourth cycle of chemotherapy demonstrated a marked reduction in metabolic activity of the penile lesion (SUVmax decreased to 2.95, Deauville score 3) (Figures 3A,D). Previously noted FDG-avid lesions in the stomach also showed interval metabolic regression (Figure 3B), with SUVmax decreasing from 6.82–7.96 (Deauville score 3) to 2.69–3.23 (Deauville score 2). No new FDG-avid lesions were identified (Figure 3C). According to the Lugano 2014 criteria and NCCN guidelines, a Deauville score of 1–3 is defined as complete metabolic response (CMR), corresponding to complete remission (CR), regardless of residual lesions. Therefore, the treatment response in this case was interpreted as complete remission.

At the completion of six cycles of chemotherapy, the patient remained clinically stable, with significant improvement in penile pain and reduction in palpable tumor size. Ongoing follow-up with oncology and urology services is planned. If disease recurrence occurs, salvage therapy with rituximab, ifosfamide, carboplatin, and etoposide (R-ICE) followed by autologous stem cell transplantation will be considered, depending on treatment response and the patient’s clinical condition.

## Discussion

Distinguishing penile lymphoma from penile carcinoma is clinically important, as treatment strategies differ substantially. While surgical intervention remains the cornerstone for penile carcinoma, systemic chemo-immunotherapy is the preferred first-line treatment for penile lymphoma, aiming to preserve penile structure and function (1–3, 7). Prior reports suggest that despite unfavorable histologic features such as non–germinal center subtype or MYC/BCL-2 double expression, DLBCL involving the penis may achieve favorable clinical and metabolic responses when appropriately treated with systemic chemo-immunotherapy (1, 2, 7).

An important diagnostic consideration is the distinction between primary penile lymphoma and secondary penile involvement in systemic lymphoma. Although primary penile lymphoma is typically defined as disease confined to the penis at presentation, the presence of additional extranodal involvement should raise suspicion for systemic disease. FDG PET/CT is the recommended imaging modality for the initial staging of FDG-avid lymphomas according to the Lugano Classification because it enables accurate whole-body assessment and detection of occult extranodal disease (4). Compared with conventional CT, PET/CT improves staging accuracy by identifying clinically unsuspected extranodal involvement, which may result in disease upstaging (5). In the present case, whole-body FDG PET/CT detected gastric involvement in addition to the clinically apparent penile lesion, allowing accurate classification as Lugano stage IV disease. Furthermore, FDG PET/CT serves as the standard modality for treatment response assessment using the Deauville five-point scale (6). Our patient achieved complete metabolic remission following R-CHOP therapy, highlighting the value of PET/CT in both baseline staging and response evaluation (Table 1).

**Table 1:** Clinical timeline of the patient.

Date	Clinical course/interventions	Results/outcome
October 15, 2025	Presented to the Urology outpatient department due to painful penile swelling and a palpable penile mass for 3 months. Physical examination revealed a hard cylindrical induration over the penile base.	Empirical antibiotic therapy was prescribed initially.
October 22, 2025	No clinical improvement after antibiotic treatment. Pelvic MRI was arranged for further evaluation.	An approximately 8 × 4.6 cm infiltrative mass involving the right corpus cavernosum and tunica albuginea was identified.
November 25, 2025	Admitted for further evaluation of progressively enlarging painful penile mass.	Initial impression was penile malignancy.
November 26, 2025	Ultrasound-guided core biopsy was performed.	Frozen-section examination revealed a malignant tumor, pending definitive histopathological diagnosis.
November 29, 2025	Permanent histopathological and immunohistochemical analyses of the penile biopsy specimen were completed.	Diffuse large B-cell lymphoma (DLBCL), centroblastic type, non-GCB subtype with MYC/BCL-2 double expression (DEL).
December 02, 2025	Readmitted for Port-A-catheter implantation, Hematology-Oncology consultation, and systemic staging workup. Whole-body FDG PET/CT was performed.	Intense FDG uptake in the penile lesion (Deauville score 5) with additional FDG-avid gastric lesion suspicious for extranodal lymphoma involvement. Curative-intent systemic chemo-immunotherapy with R-CHOP was planned.
December 03, 2025	Upper gastrointestinal endoscopy with gastric biopsy was arranged.	Gastric involvement by DLBCL was confirmed. Final diagnosis: DLBCL involving penis and stomach, Lugano stage IV.
December 05, 2025	Systemic chemo-immunotherapy with R-CHOP was initiated.	Treatment was tolerated without major complications.
March 13, 2026 After 4 cycles of R-CHOP.	Follow-up FDG PET/CT was performed for response assessment.	Marked metabolic regression of the penile lesion (SUVmax decreased to 2.95; Deauville score 3) and gastric lesion (Deauville score 2) with no newly identified FDG-avid lesions, consistent with complete metabolic response.
April 08, 2026 After 6 cycles of R-CHOP	Clinical follow-up evaluation was performed.	Significant improvement in penile pain and reduction in palpable tumor size were noted. Ongoing outpatient follow-up was planned.

Representative cases reported in recent years are summarized in Table 2 (1–3, 8–10). Most reported patients presented with localized disease (stage IE–IIE) and achieved favorable outcomes following rituximab-based chemo-immunotherapy, with surgery reserved only for selected cases. In contrast, our patient presented with advanced-stage disease (Lugano stage IV) due to synchronous gastric involvement identified by FDG PET/CT. Despite advanced-stage disease, the patient achieved complete metabolic remission following standard R-CHOP therapy, suggesting that accurate staging followed by appropriate systemic chemo-immunotherapy can result in favorable outcomes even in advanced-stage penile DLBCL.

**Table 2:** Published cases of penile diffuse large B-cell lymphoma (1–3, 8–10).

Author (Year)	Age	Stage	Extranodal site	FDG PET/CT	Treatment	Outcome	Unique feature
Fenech et al. (2)	57	IIE	None	Yes	R-CHOP ×6	Complete remission	Relatively young patient with favorable response
Diao et al. (10), Case 1*	59	IIE	None	No	R-CHOP ×6	No progression of disease	Presented mainly with penile pain
Diao et al. (10), Case 2*	79	IIE	None	No	R-CVP × 1 → R-CHOP ×6	No progression of disease	Penile erythema, swelling, ulceration
Yu et al. (1)	86	IE	None	Yes	R-COP ×2 → R-miniCHOP ×1 → subtotal penectomy	Complete remission	Surgery performed after inadequate chemotherapy response
Robertson et al. (8)	Young adult	IE	None	Yes	R-CHOP ×4	Complete metabolic response	Youngest reported patient
Fradin et al. (9)	84	IVB	Pelvis (extension to the prostate, rectum, and urinary bladder)	Yes	R-mini-CHP + polatuzumab vedotin ×6	Partial metabolic response (Deauville 4) → Deauville 1 at follow-up	Advanced-stage disease with using clonoSEQ (circulating tumor DNA) in addition to FDG PET/CT
Present case (2026)	58	IV	Stomach	Yes	R-CHOP ×6	Complete metabolic response	Synchronous gastric involvement detected by PET/CT

R-CVP, rituximab, cyclophosphamide, vincristine, and prednisone; R-COP, rituximab, cyclophosphamide, vincristine, and prednisone; R-miniCHOP, reduced-dose rituximab, cyclophosphamide, doxorubicin, vincristine, and prednisone; R-mini-CHP, reduced-dose rituximab, cyclophosphamide, doxorubicin, and prednisone. *Case 1 and Case 2 represent two individual patients reported in the same publication by Diao et al. (10).

These findings underscore the importance of comprehensive systemic evaluation, particularly with PET/CT, in patients presenting with atypical penile masses. Early tissue diagnosis through biopsy, followed by appropriate staging, is crucial to avoid misdiagnosis, prevent unnecessary surgical intervention, and ensure timely initiation of effective therapy.

This report has several limitations. First, it describes a single case, limiting the generalizability of the findings. Second, although the patient achieved complete metabolic remission after R-CHOP therapy, long-term follow-up is still ongoing, and the durability of treatment response remains to be determined. Nevertheless, this case highlights the importance of considering lymphoma in the differential diagnosis of atypical penile masses and demonstrates the value of FDG PET/CT in accurate staging and response assessment.

## Conclusion

Diffuse large B-cell lymphoma involving the penis with aches is a rare entity that may clinically mimic penile carcinoma or inflammation. This case highlights an unusual presentation of systemic DLBCL initially manifesting as a painful penile mass with advanced-stage disease. Early tissue diagnosis through biopsy and comprehensive systemic evaluation, particularly with FDG PET/CT, are essential for accurate staging, establishing the correct diagnosis, and guiding appropriate management.

## Data Availability

The datasets generated and/or analyzed during the current study are not publicly available due to patient privacy and confidentiality considerations. Requests to access the datasets should be directed to the corresponding author.
